# Machine Learning to Predict Apical Lesions: A Cross-Sectional and Model Development Study

**DOI:** 10.3390/jcm12175464

**Published:** 2023-08-23

**Authors:** Sascha Rudolf Herbst, Vinay Pitchika, Joachim Krois, Aleksander Krasowski, Falk Schwendicke

**Affiliations:** Department of Oral Diagnostics, Digital Health and Health Services Research, Charité–Universitätsmedizin Berlin, Aßmannshauser Street 4-6, 14197 Berlin, Germany; sascha.herbst@charite.de (S.R.H.); vinay.pitchika@charite.de (V.P.); joachim.krois@charite.de (J.K.); aleksander.krasowski@charite.de (A.K.)

**Keywords:** cross-sectional study, epidemiology, panoramic radiography, periapical lesions, prevalence

## Abstract

(1) Background: We aimed to identify factors associated with the presence of apical lesions (AL) in panoramic radiographs and to evaluate the predictive value of the identified factors. (2) Methodology: Panoramic radiographs from 1071 patients (age: 11–93 a, mean: 50.6 a ± 19.7 a) with 27,532 teeth were included. Each radiograph was independently assessed by five experienced dentists for AL. A range of shallow machine learning algorithms (logistic regression, k-nearest neighbor, decision tree, random forest, support vector machine, adaptive and gradient boosting) were employed to identify factors at both the patient and tooth level associated with AL and to predict AL. (3) Results: AL were detected in 522 patients (48.7%) and 1133 teeth (4.1%), whereas males showed a significantly higher prevalence than females (52.5%/44.8%; *p* < 0.05). Logistic regression found that an existing root canal treatment was the most important risk factor (adjusted Odds Ratio 16.89; 95% CI: 13.98–20.41), followed by the tooth type ‘molar’ (2.54; 2.1–3.08) and the restoration with a crown (2.1; 1.67–2.63). Associations between factors and AL were stronger and accuracy higher when using fewer complex models like decision tree (F1 score: 0.9 (0.89–0.9)). (4) Conclusions: The presence of AL was higher in root-canal treated teeth, those with crowns and molars. More complex machine learning models did not outperform less-complex ones.

## 1. Introduction

Apical lesions (AL) are a radiographic sign of a dental condition, mainly an endodontic infection [1,2]. These infections are thought to have an impact on systemic health [3] and can compromise the survival of affected teeth [4], which is why clinicians should detect and manage such lesions appropriately.

To optimize the diagnostics and treatment planning of AL, a priori knowledge on the baseline risk of a tooth or a patient suffering from AL is helpful, allowing to tailor diagnostic efforts and therapy. Cross-sectional studies based on different types of radiographs like panoramic radiographs (OPG), cone beam tomography (CBCT)) or periapical radiographs (PR) provide valuable information about the prevalence and the associated risk factors of AL. In general, prevalence of AL is assessed on two levels. (1) Patient-level prevalence is calculated by dividing the number of patients with at least one AL by the total number of examined patients. This measure describes only the positive individuals but does not take into account the number of affected teeth within the evaluated population. (2) Another important measure is the prevalence at the tooth level, which is calculated by dividing the absolute number of affected teeth by the entirety of evaluated teeth. This measure ignores possible clustering effects among patients, wherefore most studies report both measures to compensate both shortcomings.

In general, there is a broad variation among different regions in the reported prevalence of AL, from 0.6% in Norway [5] to 13.6% in Greece [6] at the tooth level. A recent systematic review and meta-analysis revealed a global prevalence of 5% at the tooth and 52% at the patient level [1]. 

The reasons for this variability are assumingly complex and manifold, for example depending on the characteristics, accessibility and education to dental care of each population. For example, Tiburcio-Machado et al., 2021 found that patients in developing countries had 2% more AL at the tooth level compared with patients of developed countries [1].

Factors concerning the prevalence of AL were identified by several studies. Lopez-Lopez et al. [7] and Sunay et al. [8] found that root-filled teeth had a significantly increased risk of AL compared with untreated teeth. 

Also, an association between sex and AL has been controversially discussed in the literature, Lopez-Lopez et al., 2012 [7] found a significant more AL in male than in female (42.3% vs. 26.1%, OR = 2.4; 95% CI (1.5, 3.7)) whereby Bürklein et al., 2020 [9] indicated no statically significant difference (47.6% vs. 39.8%, *p* > 0.05). 

Maxillary and more posterior teeth also showed a higher risk of AL compared to mandibular and more anterior teeth, respectively [10,11]. 

Additionally, Tiburcio-Machado et al., 2021 [12] demonstrated that patients with a systemic health condition had a higher prevalence of AL compared with healthy patients (48% (95% CI 43–53%; I^2^ = 98.3% vs. 63%; 95% CI 56–69%; I^2^ = 89.7%).

Most of these prevalence studies used conventional statistics and logistic regression to analyze and explain datasets and provide valuable associations between distinct variables within the respective dataset/cohort. In recent years, machine learning algorithms (MLs) are gaining strongly more popularity in the field of oral medicine [13]. With their focus on prediction rather than on explanation, MLs learn intrinsic statistical structures within datasets to eventually perform predictions on unseen data. 

So far, little is known about the predictive capacity, and, in consequence, the clinical relevance of the reported associations identified by conventional statistics and association analyses. Those predictors may help clinicians to identify important risk factors and assist the diagnostic process. Additionally, it is crucial whether the found associations were relevant and generalizable for clinical practice or if they are constrained to the internal pattern of the restricted dataset.

Therefore, we first aimed to estimate the prevalence of AL in a cohort of a German university hospital and to identify associations with a range of variables at both the patient and tooth level, respectively, using conventional statistics, logistic regression and more sophisticated MLs (k-nearest neighbor, decision tree, random forest, support vector machine as well as adaptive and gradient boosting).

Second, we yielded to utilize logistic regression and the aforementioned MLs for evaluating the predictive capacity of the found associations on the occurrence of AL on panoramic views. Hence, we tested the following hypotheses:

(1) Different MLs show no statistically significant differences with regard to their predictive performance.
(2) There is no statistically significant difference between using MLs and simply guessing the majority class of the dependent variable.

## 2. Material and Methods

### 2.1. Study Design and Source of Data

Reporting of this study follows the TRIPOD [14] and STROBE [15] guidelines as well as the checklist for artificial intelligence in dental research [16]. The study design was approved by the ethics committee of the Charité–Universitätsmedizin Berlin (EA4/080/18). Using the retrospective, cross-sectional study design, we assessed the patient records and the dental panoramic radiographs (Orthopantomogram; OPG) from the patients who presented themselves at the dental clinic of Charité University Medicine Berlin between 1 January 2015 and 31 December 2018. The device used was Sirona Orthophos XG 3 (Dentsply Sirona, York, PA, USA) and indications for taking the OPG were widely spread and were not relevant for our analyses. A formal sample size calculation was not performed, but a general rule of thumb states that the estimated sample size for logistic regression is 100 + (50 × number of independent variables in the final model) [17]. Because our study had six independent variables, the minimum sample size according to this formula was 400. All patients from pre-adolescent age with permanent teeth and over with a well conducted OPG were included in the study. The exclusion criteria for the study were patients with primary or mixed dentition, completely edentulous patients, incomplete arches and OPGs with distorted images or poor quality. In s where multiple OPGs were present in a patient, the latest one was used for the analyses. This resulted in having 1071 patients each with an OPG for the final analyses. The study sample had a mean age of 50.6 years ± 19.7 and ranged between 11 and 93 years. 

### 2.2. Image Processing and Assessment

All image data were processed in an established online annotation tool [18]. Every anatomical structure was marked pixelwise by four experienced dental radiologists. One dental radiologist reviewed all annotated OPG and evaluated each diagnosis and decided in cases of disagreement. Then, the final vote was the consensus of all annotated pixels of the radiograph. Therefore, each OPG was seen by five independent dentists eventually. Every tooth was radiologically classified by the FDI schema, restorative (fillings, crowns and root canal treatment) and apical status. Following this, the periapical status was evaluated according to the periapical index score [19]. We defined a score of at least 3 as an AL in our analysis. Based on this, we were able to calculate the prevalence of AL at both the tooth and patient level within subgroups. 

### 2.3. Variables

The covariates were divided into patient level and tooth level for presentation purposes. The patient-related information was gained from the DICOM-dataset, whereas the tooth-related information was acquired from the OPG analysis. Patients’ age (continuous variable) and gender (male or female) were patient-related variables; whereas the jaw type (upper or lower), type of the tooth (incisor, canine, premolar, or molar) and restorative status of the tooth (non-restored, filled, crown or root canal treatment) were defined as tooth-related variables.

### 2.4. Sources of Bias

The annotation process of any anatomical and pathological structure was identified as a potential source of bias. Due to the high number of examiners and a consecutive majority voting for each finding, we reduced the risk of bias in the stage of OPG analysis. 

We obtained all available radiographic data for a multivariate approach to minimize the risk of selection bias. However, we did not include clinical data, because we focused solely on the radiographical appearance of AL. Additionally, we were aware of methodological information bias resulting from the use of OPG for prevalence analysis. 

### 2.5. Statistical Analysis

First, the descriptive statistics such as number (percentage) for categorical variables and mean ± standard deviation for continuous variables for all covariates and the category-wise prevalence of apical lesions were calculated (Table 1). Second, simple bivariate comparisons of AL prevalence between the categories of the covariates were performed. Continuous and categorical variables were compared using Student’s *t*-test and a Chi-squared test, respectively. Third, a logistic regression model was constructed by regressing the patient- and tooth-level covariates for the presence (binary: present/not present) of AL. The adjusted odds ratios (aOR) and their corresponding 95% confidence intervals (95% CI) and *p*-values were calculated and tabulated (Table 2). 

**Figure 1:**
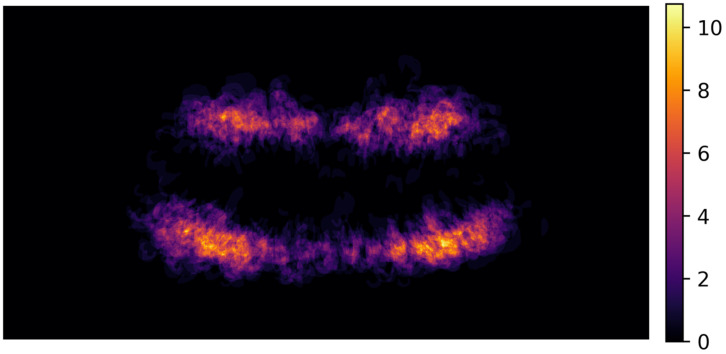
Heatmap visualizing the distribution of apical lesions. The brighter the color (yellow) indicates higher intensity in terms of more overlapping lesions; thereby, a higher number of apical lesions are detected (in total numbers).

Fourth, we trained multiple machine learning classifier models such as logistic regression, k-nearest neighbor, decision tree, random forest, support vector machine and GradientBoost, AdaBoost (Table 3) on the full dataset and evaluated the predictive performance during the 10-fold cross validation. Due to the imbalanced nature of the outcome variable, oversampling and removal of noisy data was performed with Synthetic Minority Over-Sampling Technique (SMOTE) and Edited Nearest Neighbor (ENN) for each model. During cross validation, each real sample and their synthetic correspondents as well as teeth from the same radiograph were assigned to the same split for avoiding data leakage. Balanced accuracy, precision, specificity, F1 weighted and ROC-AUC scores and their corresponding 95% CI from all machine learning models were presented (Table 4). Fifth, the no-information rate was calculated and compared with the majority class of the dependent variable AL (‘present’/’not present’) via 1-sided binomial hypothesis testing. Finally, mean rank values of the covariates based on their relative importance (based on decision tree, GradientBoost and AdaBoost models) were presented (Table 4 along with a heat map fashioned distribution of AL lesions along the dentition (Figure 1). All statistical analyses were performed using R version 4.2.2 (R Core Team 2022, Vienna, Austria); and all machine learning models were performed in Python (Version: 3.10.5). 

## 3. Results

Our study had a total of 1071 patients (male/female 51.6%/48.4%) with 27,532 teeth and with an average of 25.7 ± 6.2 teeth per patient. The mean age in our study was 50.6 ± 19.7 years, which ranged between 11 and 93 years. A total of 522 out of 1071 patients and 1133 of 27,532 teeth showed an AL, resulting in a prevalence of 48.7% at the patient level and 4.1% at the tooth level (Table 1). 

### 3.1. Statistical Analysis

We found that AL was significantly more prevalent in the patients aged ≥ 40 years than in the age group of 10–39 years; whereby men (52.5%; 95% CI: 48.4–56.7) demonstrated significantly more AL than women (44.8%; 40.5–49.1). At the tooth level, there was no significant difference between the lower (4.1%; 3.7–4.4) and upper jaw (4.2%; 3.8–4.5). Anterior teeth (incisors: 2.1%; 1.8–2.4) and canines: 2.0%; 1.6–2.5) had a significantly lower prevalence than premolars (3.7%; 3.2–4.1) and molars (7.6%; 7.0–8.2). Figure 1 shows in a heat map the distribution of AL among all OPG. We also found that non-restored teeth (1.4%; 1.2–1.6) were significantly associated with lower AL prevalence than teeth with a filling (3.3%; 2.9–3.8), a crown (4.2%; 3.6–4.8) or a root canal treatment (24.6%; 22.8–26.4) (Table 1).

The logistic regression analysis showed that at the patient level ‘male’ (aOR 1.43; 95% CI: 1.26–1.62) have higher odds to develop AL than female. In addition, it could be shown that AL occurs significantly more frequently with increasing years of life (1.01; 1.0–1.01). With respect to the tooth level covariates, the lower jaw (1.21; 1.06–1.37), molars (2.54; 2.1–3.08), teeth with fillings (1.76; 1.44–2.16), crowns (2.10; 1.67–2.63) or a root canal treatment (16.89; 13.98–20.41) had significantly higher risks of presenting with AL (Table 2). 

### 3.2. Machine Learning Models

The unbalanced tooth-level dataset (26,032 healthy surfaces vs. 1129 apical lesions) was resampled using SMOTE and ENN to arrive at a balanced dataset (22,347 healthy surfaces vs. 20,130 apical lesions). Decision tree model had the highest accuracy (0.9; 95% CI: 0.89–0.9) followed by GradientBoost (0.88; 0.87–0.88), random forest (0.87; 0.87–0.87) and k-nearest neighbor (0.87; 0.87–0.87); while logistic regression showed an accuracy of 0.83 (0.82–0.83) Compared to other models, the decision tree showed a high precision (0.9; 0.89–0.9), specificity (0.92; 0.91–0.92) and F1 score (0.9; 0.89–0.9); however, with the lowest ROC-AUC value (0.67; 0.65–0.68). 

Comparing the ROC-AUC score, logistic regression, GradientBoost, support vector machine showed statistically significant higher values compared to k-nearest neighbor, decision tree and random forest. With regard to the F1 score, the support vector machine (0.81; 0.8–0.82) significantly underperformed all other models, while the decision tree offered the highest F1 score with 0.9 (0.89–0.9). A summary of all used machine learning classifier models is given in Table 3. 

Notably, none of the model performance parameters were higher than the NIR (95.9%, *p* > 0.05). Decision tree, AdaBoost and GradientBoost assigned a score (relative importance) to each input covariate, so we calculated mean rank values for the six most important covariates (Table 4). Age was the covariate with the highest relative importance with a mean rank value of 1.7, followed by teeth restored with root canal treatments (2.3), tooth type (2.3), crowns (4.7), and sex (5.0). 

## 4. Discussion

Radiographic examinations are essential for diagnostics and treatment planning. Knowing about the prevalence and the associated risk factors of AL is helpful for evaluating the respective radiograph and sensitizes the operator to pay attention to certain regions with higher risks for AL. Our analysis aimed first to figure out which independent patient- and tooth-related variables had an influence on the dependent variable AL and second to predict the occurrence of AL based on the identified risk factors. 

### 4.1. Key Results

In our study, prevalence was similar to other studies [8,20,21,22]. One recent CBCT study from Münster/Germany found a similar prevalence of AL at the tooth level as we did [9]. Significantly higher prevalences were found in recent studies from Turkey (tooth level: 7.8%) [23], Brazil (patient level 60.5%; tooth level 8.4%), Greece (tooth level 13.6%) and Saudi Arabia (tooth level 4.5%) [24], while recent studies from Scandinavia showed a lower prevalence at the patient level (27–34%) [25,26]. A study from Finland [26], however, confirmed our findings that (1) AL is significantly more prevalent in men than in women and (2) teeth with a root canal filling are more likely associated with AL than teeth without previous endodontic treatment. In our study, root-filled teeth showed a 17-times higher probability in having an AL compared to non-treated teeth. This general finding is in line with other studies [6,7] indicating a significantly higher prevalence of AL for root canal treated teeth. A study from Denmark [27] compared two similar cohorts over ten years (1997–1998 and 2007–2009), and found out that the prevalence at both tooth and patient level has not changed significantly with time (tooth level 3.3/3.6%; patient level 42.0/45.0%); both were similar to our findings. 

We also found that restored teeth were significantly more often associated with higher AL prevalence than non-restored teeth (1.4%; 1.2–1.6), whereby the type of restoration had no statistically significant impact (filling (3.3%; 2.9–3.8), crown (4.2%; 3.6–4.8)). A cross-sectional study from Jordan [28] evaluated the periapical status of non-root-filled teeth and found also no correlation between type of restoration (composite/amalgam filling or indirect restoration) and prevalence of AL. 

The bivariate analysis showed no statistically significant difference between maxilla and mandible in prevalence in the prevalence of AL. In the multivariate analysis, it became clear that allocation to the upper or lower jaw had a significant influence on the prevalence of AL. These at a first glance contradictory results can be explained by the different utilized statistical approaches. With multivariate logistic regression analysis, we were able to examine several dependent variables for their influence on the outcome. Thus, the significance of the multivariate model exceeds that of the bivariate analysis. 

Predicting AL based on the identified risk factors was possible with differences among the selected models. Assuming that the relevance of identifying the true positive cases (AL present) is more important than the true negative class (AL not present), the F1 score is more informative for our analysis than the ROC-AUC; the F1 score is more sensitive to changes in predicting the positive class whereas the ROC-AUC balances the true negatives and the true positives [29]. The decision tree revealed the highest F1 score and outperformed the more complex models like random forest or GradientBoost. This can be explained by the straightforward associations between the identified risk factors and the presence of an AL; more complex models are intended to identify complex patterns in data structures but are more likely to fail in situations with non-complex patterns. Consequently, the identified risk factors in the logistic regression modelling offered some predictive value. When it comes to prediction accuracy, no model outperformed simply guessing the majority/negative class (“AL not present”). One reason is the high prevalence of healthy units (95.9%) at the tooth level. Overall, we had to reject the first hypothesis due to statistically significant differences in the performance of the models (F1 score/ROC-AUC) and to accept the second hypothesis, because all models did not outperform simply guessing the majority class. 

There is a difference with regard to the importance ranking of the risk factors: Multivariate analysis with logistic regression found that the presence of a root canal filling was the most important risk factor while mean rank values indicated that the patients age was most relevant. This difference might be explained by the different types of measurement scales across the models. During predictive modeling, age was categorized into nine defined age groups (Table 1) and in the multivariate analysis, age was implemented as a continuous variable. As a consequence, belonging to a distinct “age group” seems to be more significant than the gradual increase in years.

### 4.2. Limitations and Generalizability

The use of OPG for screening AL leads to information-bias, because it is known that OPG are less accurate in detecting AL than periapical radiographs (PR) or cone beam tomography (CBCT). One study found a sensitivity of 0.28 and 0.58 for OPG and PR, respectively, considering CBCT as the reference tool [30]. Also, despite technical improvements, front teeth in particular are difficult to assess in OPG, due to superimposition of anatomical structures like the cervical spine and the mental fossa area [31]. Within these limitations, OPG is still a good method in detecting AL [32]: It delivers data of the whole dentition of a patient, whereby PR and CBCT are just focusing a particular region of interest, and only a small group of patients obtain PR of all teeth, e.g., for periodontal treatment planning and we would generate an indication bias. Additionally, the indication for a CBCT of both jaws is rare, whereas the indication for OPGs is more commonly given. Hence, the group of patients obtaining an OPG is more representable than that for CBCT and full-mouth PR status. For this reason, OPGs are the most common method in cross-sectional studies [33].

In general, there is still a problem regarding the manual labeling of specialists as the ground truth for ML training [34]. For evaluating AL, histological data were still the gold standard but not available in large; thus, we tried to reduce the observer bias through a gradual majority process for labelling the AL. Every entry of each examiner was checked twice by an experienced supervisor and in case of disagreement, the experienced supervisor decided. One major advantage of this procedure is that every annotated structure and decision of PAI was transparent and saved. 

The generalizability of this study is limited due to the study design. We analyzed a local cohort from Berlin, Germany. However, based on such retrospective prevalence studies, only prevalence estimates for the entire population can be made. As mentioned before, a very recent study from another local cohort in Germany found similar prevalence values to ours [9], so the true prevalence could be in the range of our data. In general, our findings concerning the risk factors were comparable to other international studies, while they differ in the magnitude of the associations.

## 5. Conclusions

At the tooth level, posterior and restored teeth and root canal fillings showed the highest prevalence of AL. Age and sex had a significant impact on the prevalence of AL at the patient level. Predicting the occurrence of AL was possible, even though no model performed better than guessing “AL not present”. Simpler ML models outperformed more sophisticated algorithms with regard to the F1 score. 

## Figures and Tables

**Table 1:** Patient- and tooth-level characteristics and the prevalence of apical lesions and their corresponding 95% confidence intervals, stratified by the covariates in the study.

Variable	Category	N (%)/ Mean (SD)	Apical Lesions	
			N	Prevalence (95% CI)
Patient level				
All		1071 (100)	522	48.74 (45.75; 51.73)
Sex	Female	518 (48.37)	232	44.79 (40.51; 49.07)
	Male	552 (51.54)	290	52.54 (48.37; 56.70)
	Other	1 (0.09)	0	0 (0; 0)
Age	10–19 years	81 (7.56)	10	12.35 (5.18; 19.51)
	20–29 years	125 (11.67)	42	33.60 (25.32; 41.88)
	30–39 years	127 (11.86)	53	41.73 (33.16; 50.31)
	40–49 years	137 (12.79)	77	56.20 (47.90; 64.51)
	50–59 years	204 (19.05)	125	61.27 (54.59; 67.96)
	60–69 years	183 (17.09)	98	53.55 (46.33; 60.78)
	70–79 years	163 (15.22)	85	52.15 (44.48; 59.82)
	80–89 years	49 (4.58)	30	61.22 (47.58; 74.87)
	≥90 years	2 (0.19)	2	100 (100; 100)
Number of teeth		25.71 ± 6.20		
Number of fillings		7.20 ± 5.07		
Number of crowns		5.35 ± 6.11		
Number of root canal treatments		2.01 ± 2.30		
Number of apical lesions		1.06 ± 1.56		
Tooth level				
Tooth status	Present	27,532 (80.33)	1133	4.12 (3.88; 4.35)
	Missing	6740 (19.67)		
Jaw	Upper	13,519 (49.10)	564	4.17 (3.83; 4.51)
	Lower	14,013 (50.90)	569	4.06 (3.73; 4.39)
Type of tooth	Incisor	7948 (28.87)	165	2.08 (1.76; 2.39)
	Canine	4083 (14.83)	83	2.03 (1.60; 2.47)
	Premolar	7394 (26.86)	270	3.65 (3.22; 4.08)
	Molar	8107 (29.45)	615	7.59 (7.01; 8.16)
Restorative status	Non-restored	14,572 (52.93)	209	1.43 (1.24; 1.63)
	Filling	6409 (23.28)	213	3.32 (2.88; 3.76)
	Crown	4403 (15.99)	183	4.16 (3.57; 4.75)
	Root canal treatment	2148 (7.80)	528	24.58 (22.76; 26.40)

Descriptive statistics were presented as number and percentages N (%) for categorical variables, and as mean (standard deviation (SD)) for continuous variables.

**Table 2:** Logistic regression analysis of the associations between the presence of apical lesions and covariates. The results are presented as adjusted odds ratios, 95% confidence intervals and their corresponding *p*-values at patient and tooth level, respectively. Statistically significant associations are indicated in bold.

Covariate	Category	Logistic Regression	
		Adjusted Odds Ratio (95% CI)	*p*-Value
Patient level			
Sex	Female (Ref.)	-	-
	Male	1.43 (1.26; 1.62)	<0.0001
Age	-	1.01 (1.00; 1.01)	0.0006
Tooth level			
Jaw	Upper jaw (Ref.)	-	-
	Lower jaw	1.21 (1.06; 1.37)	<0.005
Tooth	Incisor (Ref.)	-	-
	Canine	0.94 (0.72; 1.24)	0.67
	Premolar	1.18 (0.96; 1.45)	0.12
	Molar	2.54 (2.10; 3.08)	<0.0001
Restoration status	Non-restored (Ref.)	-	-
	Filling	1.76 (1.44; 2.16)	<0.0001
	Crown	2.10 (1.67; 2.63)	<0.0001
	Root canal treatment	16.89 (13.98; 20.41)	<0.0001

**Table 3:** Summary of machine learning models. Oversampling and removing of noisy data performed with SMOTE and ENN.

Classifier	Accuracy (95% CI)	Precision (95% CI)	Specificity (95% CI)	F1 (95% CI)	ROC-AUC (95% CI)
Logistic regression	0.83 ^a^ (0.82; 0.83)	0.83 ^a^ (0.82; 0.83)	0.84 ^a^ (0.83; 0.84)	0.83 ^a^ (0.83; 0.84)	0.79 ^a^ (0.77; 0.80)
k-nearest neighbor	0.87 ^b^ (0.87; 0.87)	0.87 ^b^ (0.87; 0.87)	0.89 ^b^ (0.88; 0.89)	0.87 ^b^ (0.87; 0.87)	0.70 ^b^ (0.68; 0.72)
Decision tree	0.90 ^c^ (0.89; 0.90)	0.90 ^c^ (0.89; 0.90)	0.92 ^c^ (0.91; 0.92)	0.90 ^c^ (0.89; 0.90)	0.67 ^c^ (0.65; 0.68)
Random forest	0.87 ^b^ (0.87; 0.87)	0.87 ^b^ (0.87; 0.87)	0.89 ^b^ (0.88; 0.89)	0.87 ^b^ (0.87; 0.87)	0.71 ^b^ (0.69; 0.72)
Support vector machine	0.81 ^d^ (0.80; 0.82)	0.81 ^d^ (0.80; 0.82)	0.82 ^d^ (0.81; 0.82)	0.81 ^d^ (0.80; 0.82)	0.78 ^a^ (0.77; 0.80)
GradientBoost	0.88 ^f^ (0.87; 0.88)	0.88 ^b^ (0.87; 0.88)	0.89 ^b^ (0.89; 0.89)	0.88 ^b^ (0.87; 0.88)	0.80 ^a^ (0.79; 0.82)
AdaBoost	0.84 ^g^ (0.83; 0.84)	0.84 ^e^ (0.83; 0.84)	0.85 ^a^ (0.84; 0.85)	0.84 ^a^ (0.83; 0.84)	0.80 ^a^ (0.79; 0.82)

^a–g^ different letters indicate statistically significant differences (*p* < 0.05).

**Table 4:** Mean rank values based on relative importance (decision tree, AdaBoost, GradientBoost).

Feature	Mean Rank Value
Age	1.7
Root canal treatment	2.3
Tooth type	2.3
Crowns	4.7
Sex	5.0

## Data Availability

The data are available upon request from the corresponding author.
